# *Notes from the Field*: Initial Outbreak Response Activity Following Wild Poliovirus Type 1 Detection — Malawi, February 2022

**DOI:** 10.15585/mmwr.mm7123a3

**Published:** 2022-06-10

**Authors:** Malawi Ministry of Health, Global Polio Eradication Initiative, Elizabeth Davlantes

**Affiliations:** 1 Global Immunization Division, Center for Global Health, CDC.

Since the Global Polio Eradication Initiative (GPEI) began in 1988, the number of wild poliovirus (WPV) cases has decreased by >99.99%, and five of the six World Health Organization (WHO) regions are now certified WPV-free.* WPV serotypes 2 and 3 have been declared eradicated (*1*), and WPV type 1 (WPV1) is currently endemic only in Pakistan and Afghanistan in the WHO’s Eastern Mediterranean Region (*2*,*3*).

The WHO African Region was certified free of indigenous WPV transmission on August 25, 2020 (*4*). Approximately 18 months later on February 16, 2022, a paralytic WPV1 case was confirmed in a child aged 3 years residing in Lilongwe, Malawi, in southeastern Africa, with paralysis onset November 19, 2021. The affected child had no history of travel or contact with anyone who had traveled internationally and had received only 1 of 5 doses of poliovirus vaccine recommended by the Malawi Ministry of Health through routine childhood immunization services. Genomic sequence analysis of the isolated poliovirus indicated that its closest relative was a WPV1 lineage isolated from samples taken in Sindh Province, Pakistan, in October 2019. Before this detection in Malawi, the last WPV1 case in Africa had been reported in Nigeria in 2016 (*4*).

Within 24 hours of virus identification, the president of Malawi declared a public health emergency and activated the country’s emergency operations center. Within 3 days of case confirmation, a team of GPEI partners had arrived in Malawi to support the Ministry of Health in strengthening acute flaccid paralysis surveillance, reeducating local clinicians and public health professionals, and organizing nationwide outbreak response supplementary immunization activities (SIAs) to reach 2.9 million children aged <5 years with bivalent oral poliovirus vaccine (bOPV, containing Sabin strain serotypes 1 and 3). The first nationwide bOPV outbreak response SIA began on March 21, 2022, and additional nationwide SIAs are planned over the coming months.

In addition, GPEI has engaged with the countries surrounding Malawi to increase their preparedness for potential cross-border spread of the virus. Until polio is eradicated worldwide, all countries must be vigilant against importation of polio and reestablishment of local transmission. GPEI teams have worked closely with the Ministries of Health in Mozambique, Tanzania, and Zambia to strengthen surveillance and support implementation of subnational bOPV SIAs in areas bordering Malawi (Figure). The coordinated campaigns began on March 24, 2022, targeting 6.4 million children aged <5 years. Subsequent nationwide SIAs are planned in these neighboring countries, as well as in Zimbabwe.

**FIGURE:**
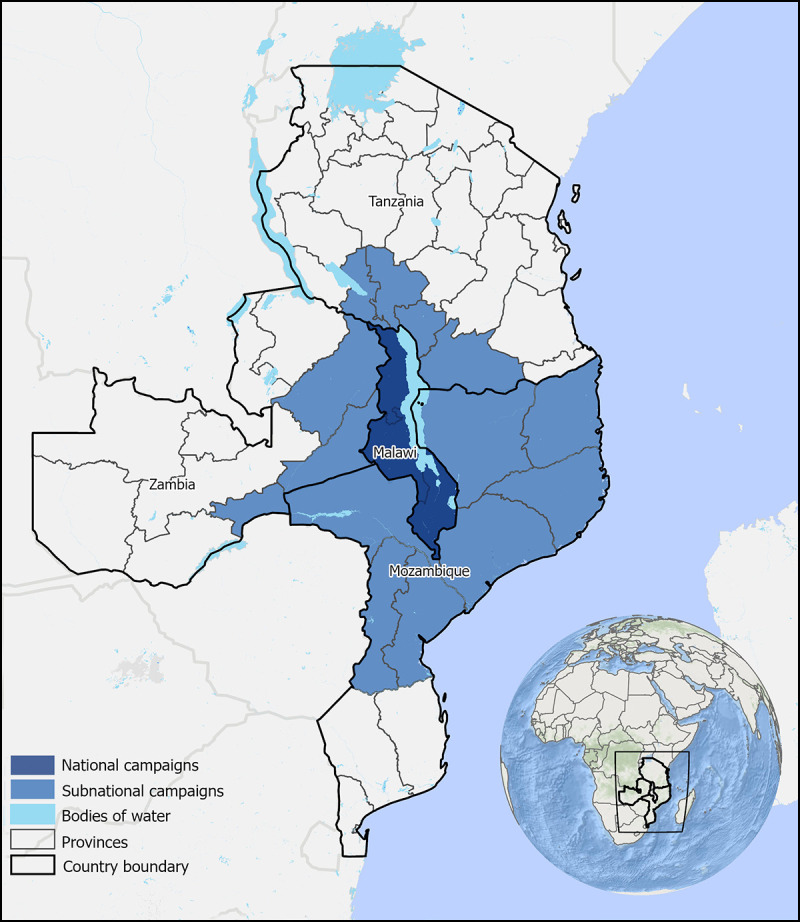
Multicountry outbreak response supplementary immunization activity (round 1) in response to a case of wild poliovirus type 1 — Malawi, 2022

An additional case of paralytic WPV1 was detected in Tete Province, Mozambique, on April 1, 2022, with paralysis onset March 25, 2022. Existing response efforts are being modified to address this case in addition to the case in Malawi.

## Footnotes

*
https://www.cdc.gov/polio/progress/index.htm#:~:text
